# Ultrasonographic Measurement of Skin to Lumbar Epidural Space Depth in Pregnant Women Undergoing Elective Cesarean Section: An Observational Study

**DOI:** 10.31729/jnma.8861

**Published:** 2025-01-31

**Authors:** Sushila Lama Moktan, Milan Kumar Thapa, Ujma Shrestha, Prabin Subedi

**Affiliations:** 1 Department of Anesthesia and Intensive Care, Kathmandu Medical College Teaching Hospital, Sinamangal, Kathmandu, Nepal; 2 Department of Anesthesia and Intensive Care, Grande International Hospital, Tokha, Kathmandu, Nepal

**Keywords:** *lumbar space*, *obstetrics*, *ultrasound*

## Abstract

**Introduction:**

Neuraxial anesthesia is commonly used during cesarean delivery and is preferred for its effectiveness and minimal risks. Accurate identification of the depth of the lumbar epidural space is crucial to ensure successful block and minimize complications. However, this can be challenging in pregnant women due to physiological changes that affect the anatomy of the lumbar spine. Ultrasound is a helpful device for providing real-time visualization of the relevant anatomical structures and facilitating accurate needle insertion. In this study, we evaluated the utility of ultrasound in determining the depth from the skin to the lumbar epidural space in pregnant women undergoing elective caesarean section.

**Methods:**

This is an observational cross-section study performed after approval from Institutional Review Committee (Reference number:09062023/03). Using a curvilinear ultrasound probe, imaging of spine at lumbar third and fourth intervertebral space was performed. The measurements were performed in the sitting position with the patients' backs flexed in the parasagittal oblique and transverse median view to determine the distance from the skin to the lumbar epidural space.

**Results:**

The mean depth from the skin to the lumbar epidural space/posterior complex in the parasagittal oblique view was 46.84±7.18 mm (95% CI : 45.48-48.20), and transverse median view was 45.27±8.16mm (95% CI :43.73-46.81).

**Conclusions:**

The skin-to-lumbar epidural space depth in pregnant women undergoing elective cesarean section was comparable to other studies conducted in similar settings.

## INTRODUCTION

Neuraxial ultrasonography (USG) has grown in popularity due to its ease of use, ability to accurately localize and visualize the spinal anatomy, resulting in improved safety and lower rate of complications.^1^

Conventional palpation method is often challenging and ambiguous to localize the desired intervertebral space,^2-4^ because of weight gain, oedema, and other physiological changes related to pregnancy in the obstetric population.^5-7^ The difficulty in locating surface anatomical landmarks and variability in distance from skin to epidural space prevents it from being correctly identified.^3,8^ USG is often used throughout the pregnancy, thus parturient are acquainted with it and has no detrimental effects on the mother or foetus. It is a transportable device that is easily accessible in operation theatre.

The goal of this study is to use USG to measure the distance to the epidural space from skin prior the performance of spinal anaesthesia.

## METHODS

This observational cross-section study was conducted at Kathmandu Medical College Teaching Hospital, a tertiary care hospital located at Sinamangal, Kathmandu, Nepal. The study was conducted from August 2023 to July 2024 And data was collected after receiving ethical approval from the Institutional Review Committee (Reference number: ). The study population was parturient females scheduled for cesarean section. The sample size was calculated using the following formula:

Sample size


$$\begin{array}{ll} \text{n} & ={\text{Z}}^{2}\times \frac{\sigma^{\text{2}}}{{\text{e}}^{\text{2}}}\\ & =1.96^{2}\times \frac{0.77^{2}}{0.154^{2}}\\ & =100 \end{array}$$

10% drop out of the study participant: 110
n = Desired number of samples
Z1-α/2 = 1.96 at 95% confidence interval
d (Margin of error) = 0.154
σ (SD which is based on previous study) = 0.7711^9^

The estimated sample size was 110 and the purposive sampling was done. All parturient with the American Society of Anaesthesiologists (ASA-PS) physical status II-III, singleton term pregnancies (37-42 weeks of gestation) aged 18-40 years and providing consent were included in the study. While, parturients with neurological diseases, vertebral column anomalies, history of spine or spinal canal surgeries, ASA-PS >III, multiple pregnancies, complicated pregnancies, emergency cesarean sections, coagulopathy or history of anticoagulant medication use, and patients rejecting spinal anesthesia were excluded from the study.

Written, informed consent was taken from the after ensuring that the participants has understood the details provided in the information sheet. The patient underwent scan using a GE LOGIQ TM e ultrasound machine, conducted by a different investigator who had knowledge and experience with USG imaging of spine. A curved array probe (2-5MHz) was used for scanning process in an unsterile manner. The distance from skin to posterior complex (ligamentum flavum, epidural space and posterior dura) recorded in millimeters(mm) was accepted as the epidural space depth (ED) in both the parasagittal oblique (PSO) plane and transverse median (TM) plane. For PSO plane measurement the probe was aligned vertically parallel to the long axis of the spine. The device was initially positioned over the sacral region, 1-2 cm lateral to the midline and angled slightly to target the midline of the spine. This allowed the user to visualize a continuous bright, hyperechoic line that represented the sacrum, then the probe was gradually moved in the cephalad direction to locate a hyperechoic image that resembled a "sawtooth". Keeping the image in view, the probe was advanced further cephalad until it reached third and fourth lumbar(L3-L4) intervertebral space. The most hyperechogenic structure visible posteriorly is the posterior complex . Using the US devices integrated caliper, the monitor was frozen to measure the skin to epidural depth. In order to determine the L3-L4 intervertebral space in the horizontal plane, the skin was marked on both sides of the probe at midline. The TM plane measurement was done by rotating the probe to view in transverse median plane on predetermined horizontal line at the L3-L4 intervertebral space. The spinous process was visualized by moving the probe either cephalad or caudad the skin was marked on both sides of the probe in the midline. The probe was angled upward or downward until a desired picture of "batwing" appearance was obtained. Once the posterior complex was clearly visible, the pressure on the probe was gradually decreased to lessen the pressure applied on the soft tissue until the anatomical structures are still legibly visible then the monitor was frozen for the measurement in TM. A numerical scoring system was also used to assess the visibility of the posterior complex in TM view under ultrasonography (0= none, 1= hardly, 2= well, 3= very well detectable).^10^

Each patients basic information including age, height, weight, body mass index (BMI), parity, gestational age, indication for caesarean section was recorded. Patients were made to sit with neck flexed, arms resting on the side and feet resting on the tool in the operation theater. One of the anesthesiologists who was not involved in scanning performed the lumbar area examination using the spinal landmark grade system (SLGS) Grade 1=visible spinous processes; grade 2 = spinous processes are not seen but easily palpated; grade 3 = spinous processes are not seen and not palpated but the interval between them is palpated as a low landmark under the thumb; grade 4 = none of the previous cases.^10^

The data were collected and entered in Microsoft Excel and then transferred to IBM SPSS Statistics for Windows, version 26 (IBM Corp., Armonk, N.Y., USA) for analysis. Data were represented as mean and standard deviation (SD) and point estimation at a 95% confidence interval (CI) was calculated.

## RESULTS

A complete lumbar neuraxial USG examination was performed on 110 patients. In the PSO view the distance from skin to epidural space was 46.84±7.18 mm (95% CI: 45.48-48.20), and in TM view it was 45.27±8.16mm (95% CI: 43.73-46.81). The mean age of parturient was 30.85±3.85, mean BMI was 30.68±3.71, (Table 1).

**Table 1:** Physical characteristics of parturient undergoing neuraxial ultrasound examination (n=110).

Characteristics	n (%)
Age (years)	30.85±3.85
Height (cm)	154.73±5.07
Weight (kg)	73.67±9.57
BMI (kg/m^2^)	30.68±3.71
<30	37(33.63%)
>30	73(66.36%)
Gestational age (weeks)	38.13±0.76

The history of previous CS was the indication for CS in 70 (63.36%) parturients, the cephalo-pelvic disproportion in 18 (16.40%), and miscellaneous (breech presentation, bad obstetric history, maternal request, medical conditions) in 22 (20.34%) of parturients. Co-morbidities were present in 56 (50.90%) of the patients, of which 18 (16.36%) had hypothyroidism, 16 (14.54%) had gestational diabetes mellitus, and remaining had variety of illnesses like hypertension, obstetric cholestasis, cardiac diseases and Hepatitis B positive status.

Observation of the anatomical characteristics of the spine using SLGS revealed that 65 (59.09%) had grade 2 spinal landmark type (Table 2).

**Table 2:** Spinal landmark grading system (n=110).

Landmark	n (%)
1= spinous processes are visible	14(12.72)
2= spinous processes are not seen but easily palpated	65(59.09)
3= spinous processes are not seen and not palpated but interval between them is palpated as low landmark under the thumb	29(26.36)
4= none of the previous cases	2(1.81)

The visibility of the posterior complex, which is the major target image of ultrasonography, was detected in all the patients, and was well detectable in 85 (77.27 %) parturient, (Table 3).

**Table 3:** Visibility of posterior complex in ultrasound images (n=110).

Visibility of posterior complex	n (%)
0 (none)	-
1 (hardly)	8 (7.27)
2 (well)	85 (77.27)
3 (very well)	17 (15.45)

## DISCUSSION

Ultrasound imaging of the lumbar spine was performed in 110 parturient and the distance from the skin to epidural space/posterior complex was measured. The mean distance in PSO view was 46.84±7.18 mm, and TM view was 45.27±8.16mm in the L3-L4 intervertebral space. Similar to our study, the average length of the perpendicular trajectory from the skin surface to the ligamentum flavum was 49.5 (8.1) mm.^12^ The skin-to-Epidural space distance range in a 3011 obstetric patients was studied by Sutton and Linter where merely 76% fell within a ''normal'' range (4-6 cm).^13^ The US estimate in the PSO and TM planes for the study of obese parturient with mean BMI of 30.6 kg/m^2^ was 6.5 (1.2) cm and 6.5 (1.1) cm respectively.^14^ The mean distance measured by Kanwat in Indian parturient of 26 kg/m^2^ BMI was 3.61 cm±0.17 cm.^15^ With the use of ultrasonography these variations in the distance between skin to the epidural space can be accurately identified.

Regarding the SLGS in our study, 65 (59.09%) of the patients had grade 2 and 29 (26.36%) had grade 3. In a prior Taiwanese study, 63.3% of patients had spinal landmark grade of 2 and 16.7% had grade 3 compared to only 3.3% of non-pregnant population had grade 3.^10^ According to the study by Chin, preprocedural ultrasound imaging helps with spinal anaesthesia in the non-obstetric patient population with challenging anatomic landmarks with higher grades of SLGS. grade 3 in 43%, and grade 4 in 18% in a patient with BMI >35kg/m^2^.^16^ A strong positive association was observed between SLGS and lumbar puncture grading; that is, in patients where spinous process was not visible or palpable had difficult lumbar puncture.^17^

In our study, the posterior complex as a target structure was visualized well in 77.27%, and very well in 15.45% of cases. Of the 36 patients in the anticipated cases of difficult epidural anesthesia, 25 (69.44%) had good visibility of ligamentum flavum, 8 (2.22%) had sufficient visibility and 3 (8.34%) had none at all.^5^ In another study, the visibility of the ligamentum flavum in pregnant women was rated as "very well detectable". Pregnancy affects the quality of sonographic depiction of the key structures. However, ultrasonography can serve as a useful guide for parturient undergoing neuraxial block. Although the average quality is depleted by 50-70% in pregnancy than in non-pregnant state, it was still sufficient to identify the ligamentum flavum and the epidural space in all cases.^12^

Neuraxial block procedure is technically demanding, particularly in obstetric setting as spinal anatomy is significantly altered by pregnancy related changes like weight gain, increased subcutaneous adipose tissue, oedema, loosening of the ligaments and soft tissue, hyper lordosis and progressive pelvic rotation.^5,6^ It often takes several attempts to find the space because of these anatomical changes and raises the possibility of complications. Many factors can contribute to regional anaesthesia complications and failures, but the primary one being the blind nature of the technique. Palpation is the only method established to localize the intervertebral space during neuraxial block. The ways to measure the depth other than ultrasonography are by can lateral X-ray of the lumbar spine or computerized axial tomography, but radiography is not an appropriate diagnostic tool during pregnancy. Magnetic resonance imaging has expanded the diagnostic capabilities,^18^ but is also impractical in labour. The alterations of spinal anatomy associated with pregnancy were readily observed by the use of ultrasound imaging.^12^ Strong correlations between actual depth measured by epidural needle and the depth estimated by ultrasound using both PSO and TM view have been documented in number of previous studies.^6,19,20^

The study has a number of limitations. First, the epidural space depth was measured perpendicularly without taking the oblique trajectory into account so that the real depth might be underestimated. Second, the actual needle depth was not measured therefore, the real epidural depths could not be compared. Furthermore, the study was not conducted in patients with difficult anatomy like spinal deformities, meaning it might not accurately reflect the other challenging patients.

Lumbar spine ultrasound scanning can provide an accurate measurement of the depth to the extradural space from skin. Knowing the depth a head of time should make it easier to perform the neuraxial block and potentially lower the rate of complication.

## CONCLUSIONS

The skin-to-lumbar epidural space depth in pregnant women undergoing elective cesarean section was comparable to other studies conducted in similar settings. Most of the patients included in our study had obesity grade I or higher and the as per spinal landmark grading system spinous process are not seen but easily palpable.

## References

[1] Teissier H, Lozano C, Plavsic KS (2014). The Use of Ultrasonography for the Guidance of Epidural Analgesia in Obstetric Anesthesia.. Donald Sch J Ultrasound Obstet Gynecol..

[2] Borges BC, Wieczorek P, Balki M, Carvalho JC (2009). Sonoanatomy of the Lumbar Spine of Pregnant Women at Term.. Reg Anesth Pain Med..

[3] Broadbent CR, Maxwell WB, Ferrie R, Wilson DJ, Gawne-Cain M, Russell R (2000). Ability of Anesthetists to Identify a Marked Lumbar Interspace.. Anaesthesia..

[4] Whitty R, Moore M, Macarthur A (2008). Identification of the Lumbar Interspinous Spaces: Palpation Versus Ultrasound.. Anesth Analg..

[5] Grau T, Leipold RW, Conradi R, Martin E (2001). Ultrasound Control for Presumed Difficult Epidural Puncture.. Acta Anaesthesiologica Scandinavica..

[6] Grau T, Leipold RW, Conradi R, Martin E, Motsch J (2002). Efficacy of Ultrasound Imaging in Obstetric Epidural Anesthesia.. J Clin Anesth..

[7] Sahin T, Balaban O, Sahin L, Solak M, Toker K (2014). A Randomized Controlled Trial of Preinsertion Ultrasound Guidance for Spinal Anaesthesia in Pregnancy: Outcomes Among Obese and Lean Parturients: Ultrasound for Spinal Anesthesia in Pregnancy.. J Anesth..

[8] Furness G, Reilly MP, Kuchi S (2002). An Evaluation of Ultrasound Imaging for Identification of Lumbar Intervertebral Level.. Anaesthesia..

[9] Lu IC, Huang SH, Hsu CD, Chiu CH, Wu SH (2011). Lumbar Epidural Space Was Narrower in Parturients Than That in Nonpregnant Women by Ultrasound Assessment.. Kaohsiung Journal of Medical Sciences..

[10] Chien I, Lu IC, Wang FY, Soo LY, Yu KL, Tang CS (2003). Spinal Process Landmark as a Predicting Factor for Difficult Epidural Block: A Prospective Study in Taiwanese Patients.. Kaoshiung J Med Sci..

[11] Capogna G, Baglioni S, Milazzo V, Vitale A (2018). Accuracy of the SpineNav3DTM Technology to Measure the Depth of Epidural Space: A Comparison with the Standard Ultrasound Technique in Pregnant Volunteers.. Open Journal of Anesthesiology.

[12] Grau T, Leipold RW, Horter J, Conradi R, Martin E, Motsch J (2001). A Lumbar Epidural Space in Pregnancy: Visualization by Ultrasonography.. British Journal of Anaesthesia..

[13] Sutton DN, Linter SPK (1991). Depth of Epidural Space and Dural Puncture.. Anaesthesia..

[14] Sahota JS, Carvalho JCA, Balki M, Fanning N, Arzola C (2013). Ultrasound Estimates for Midline Epidural Punctures in the Obese Parturient: Paramedian Sagittal Oblique Is Comparable to Transverse Median Plane.. Anesth Analg..

[15] Kanwat J (2021). To Study the Correlation between Estimated Ultrasound Depth and Actual Needle Depth of Epidural Space in Transverse Plane in Parturients Undergoing Cesarean Section.. J Anesth Clin Res..

[16] Chin KJ, Perlas A, Vincent Chan, Brown-Shreves D, Koshkin A, Vaishnav V (2011). Ultrasound Imaging Facilitates Spinal Anesthesia in Adults with Difficult Surface AnatomicLandmarks.. Anesthesiology..

[17] Subramanian S, Reshma BM, Iqbal MS, Harsoor SS (2023). A Comprehensive, Bed-Side Scoring System to Predict Difficult Lumbar Puncture.. J Anaesthesiol Clin Pharmacol..

[18] Crighton IM, Barry BP, Hobbs GJ (1997). A Study of the Anatomy of the Caudal Space Using Magnetic Resonance Imaging.. British Journal of Anaesthesia..

[19] Balki M, Lee Y, Halpern S, Carvalho JCA (2009). Ultrasound Imaging of the Lumbar Spine in the Transverse Plane: The Correlation Between Estimated and Actual Depth to the Epidural Space in Obese Parturients.. Anesth Analg..

[20] Canturk M (2019). Correlation of Actual Epidural Depth With Ultrasound Estimates in Paramedian Sagittal Oblique and Transverse Median Plane in Parturients: A Prospective Cross-Sectional Study.. Int J Clin Exp Med..

